# Infection effects of the new microsporidian species *Tubulinosema suzukii* on its host *Drosophila suzukii*

**DOI:** 10.1038/s41598-021-89583-9

**Published:** 2021-05-12

**Authors:** Sarah Biganski, Sabrina Fückel, Johannes A. Jehle, Regina G. Kleespies

**Affiliations:** 1 Federal Research Centre for Cultivated Plants, Institute for Biological Control, Julius Kühn Institute, Heinrichstraße 243, 64287 Darmstadt, Germany; 2 Technische Universität Darmstadt, Schnittspahnstraße 10, 64287 Darmstadt, Germany

**Keywords:** Ecology, Ecological epidemiology

## Abstract

Microsporidian infections of insects are important natural constraints of population growth, often reducing lifespan, fecundity and fertility of the infected host. The recently discovered *Tubulinosema suzukii* infects *Drosophila suzukii* (spotted wing drosophila, SWD)*,* an invasive pest of many fruit crops in North America and Europe. In laboratory tests, fitness effects on larval and adult stages were explored. High level infection after larval treatment caused up to 70% pupal mortality, a decreased lifespan and a 70% reduced oviposition of emerging adults in biparental infection clusters. A shift to higher proportion of female offspring compared to controls suggested a potential parthenogenetic effect after microsporidian infection. A clear sex-linkage of effects was noted; females were specifically impaired, as concluded from fecundity tests with only infected female parents. Additive effects were noted when both parental sexes were infected, whereas least effects were found with only infected male parents, though survival of males was most negatively affected if they were fed with *T. suzukii* spores in the adult stage. Although most negative effects on fitness parameters were revealed after larval treatment, infection of offspring was never higher than 4%, suggesting limited vertical transmission. For that reason, a self-reliant spread in natural SWD populations would probably only occur by spore release from cadavers or frass.

## Introduction

Microsporidia are spore-forming, obligate intracellular parasites early evolved presumably as a basal linage or a sister taxon to Fungi^1–4^. They infect a huge range of arthropod species but also vertebrates including humans^5^. Oral ingestion of spores is the major form of horizontal transmission where spores enter the host cell cytoplasm by penetration of midgut cells after everting the polar tube. Other infection routes via vertical transmission are either transovarial: inside egg or embryo^6^, *transovum*: on the surface of the egg chorion^7,8^, or venereal transmission: paternal^9^. To reproduce and spread within the host tissue, microsporidia undergo several morphological changes from meronts (proliferation) to spores (sporogony)^8,10^. Severe infections located in one or more host tissues or organs can lead to highly negative health effects on the host, as it is known for *Nosema* disease in honeybees causing heavy intestine disorders, shortened lifespan and neuronal/behavioural changes leading to disorientation^11–15^. Though, microsporidia are rarely fast-killing pathogens, they are able to reduce host populations by weakening the host fitness such as reduction of fertility and offspring, growth rate, and life span. Horizontally transmitted microsporidia frequently show higher virulence than vertically transmitted, and is commonly favoured by high host population density, e.g. honeybees and gypsy moth populations^16,17^. Vertical transmission is frequently found with microsporidia with low virulence, depending on host survival, fertility and number of infected offspring to ensure their own reproduction^18,19^. Vertical transmission has been noted for at least fourteen Microsporidia taxa^20^ and is known to reduce fecundity and fertility in several insect hosts^21–24^. Although there are some microsporidian species that increase mortality of their insect host, their usage as pest control agent in plant protection has been rarely considered. *Paranosema* (*Nosema*) *locustae*, infecting acridids, is an example of efficient but also problematic use of microsporidia in biological control (reviewed by Lockwood et al.^25^).

Due to human activity, travel and worldwide transport of agricultural products, but also climate change, the spread of pest insects of cultivated plants has been highly facilitated, resulting in the invasion of new pests and diseases to previously unaffected regions^26^. In about a decade the spotted wing Drosophila (SWD), *Drosophila suzukii*^27^, has become a major pest in commercial soft-skinned fruit orchards in Europe, North and South America and Asia^27–29^. Naturally occurring antagonists, both metazoans^30,31^ and microorganisms, may play an important role for biological control of SWD but efficient microbial antagonists have not been reported^31–33^. Antagonistic fungi and bacteria have been screened but also criticized for inefficiency and/or inappropriate application^33–35^. In addition, some viruses have been identified from SWD that should be examined for their potential as biocontrol agents^36,37^.

Recently, a microsporidian infection was discovered in SWD flies originating from Oregon, USA^38^ and a new species *Tubulinosema suzukii* (family: Tubulinosematidae) was described^39^. Here, we report laboratory experiments to explore the potential effect of *T. suzukii* infection on fitness parameters of SWD, including eclosion and survival rates, egg production and offspring rates, when infections were initiated in larval and adult stages. We found that every fitness parameter tested was strongly impacted when infection began in the larval stage.

## Results

### Median lethal concentration (LC_50_)

**Figure 1:**
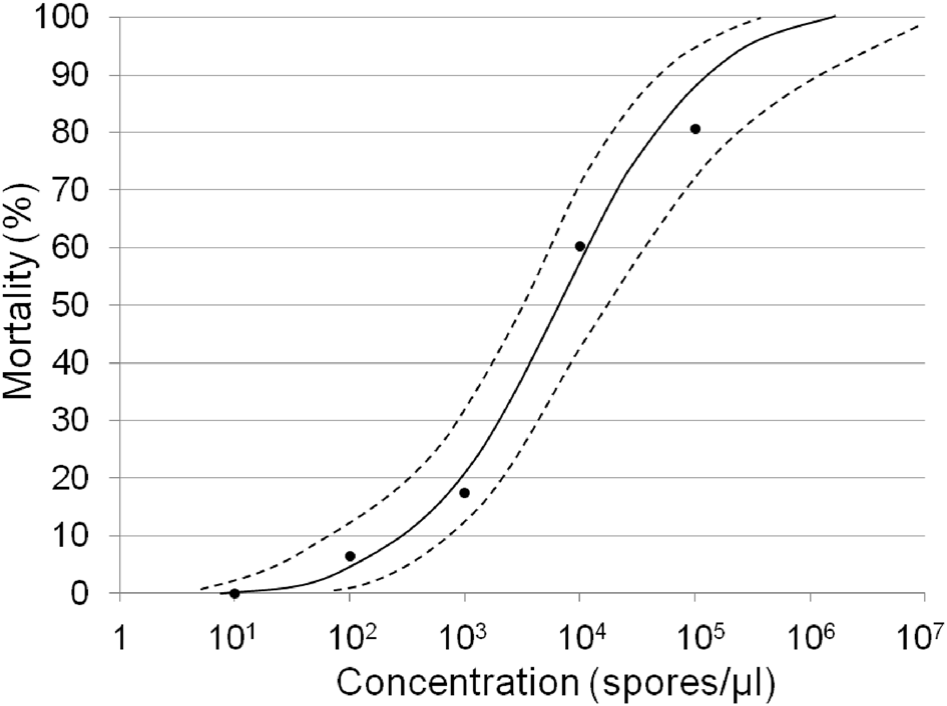
Spore concentration-mortality response of *D. suzukii* second instar larvae, 18 days after exposure to *T. suzukii* spores. Independent replicates/number of tested individuals (R/N): untreated control: R/N = 9/252, 10^1^ spores: R/N = 2/96, 10^2^ spores: R/N = 5/120, 10^3^ spores: R/N = 6/180, 10^4^ spores: R/N = 7/211, 10^5^ spores: R/N = 2/24). Logarithm Log(x) of concentrations and number of organisms entered the probit analysis using linear maximum likelihood regression. Slope function was calculated after Litchfield and Wilcoxon. Concentration–response: *F* = 76.966, *DF* = 3, *P*(F) = 0.003. Dots = observed mortality, solid line = calculated concentration, dashed lines = upper and lower 95% confidence limits.

Inoculation of second instar SWD larvae (L2) with suspensions of five different spore concentrations of *T. suzukii* resulted in a LC_50_ of approximately 6.9 × 10^3^ spores/μl (95% confidence limits (CL) = 3.4 × 10^3^–1.7 × 10^4^ spores/μl, N = 631, slope = 0.915, *χ*^2^ = 21.683) (Fig. 1). Mortality rates were determined by failure of the emergence of imagines because death of larvae could not be determined within the growth medium. Control mortality was 28.2% after 18 days post inoculation (dpi). Maximum mortality was 80% at the highest spore concentration applied.

### Quantification of infection process

**Figure 2:**
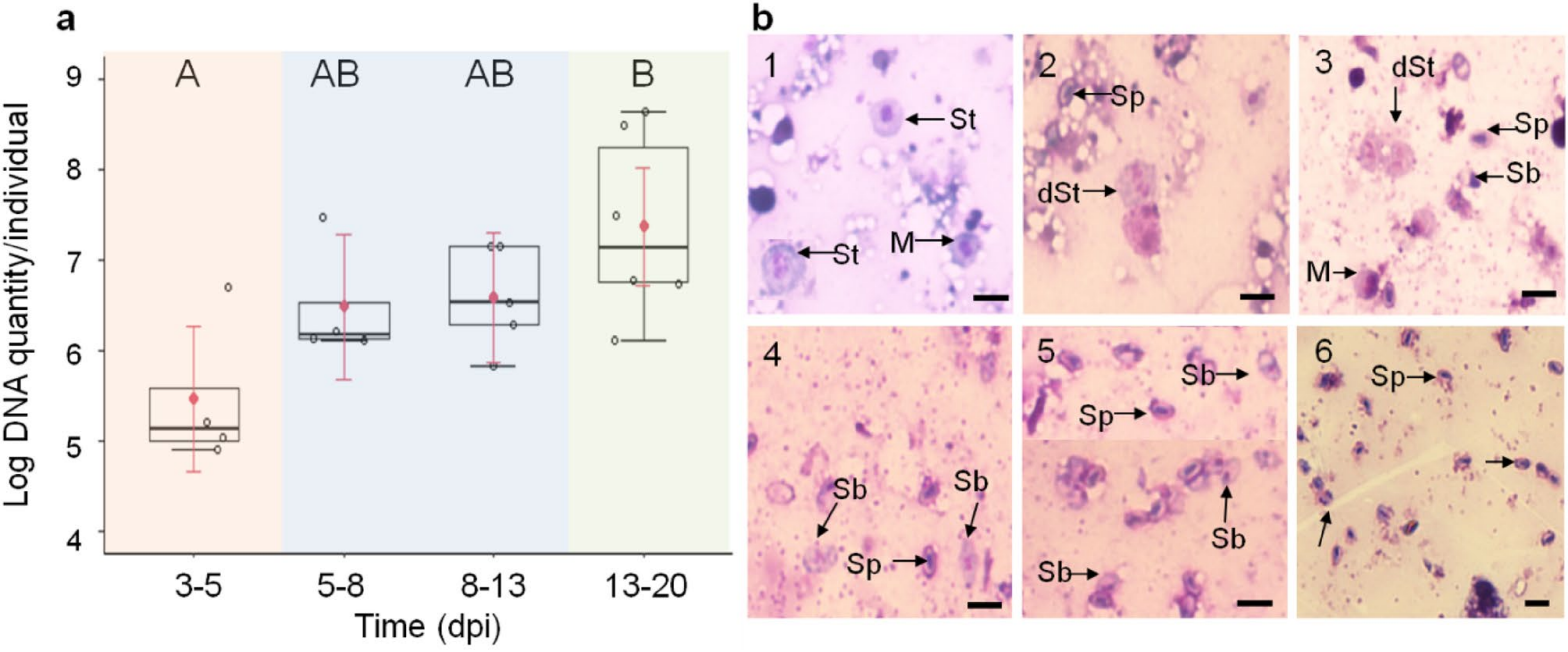
(**a**) Copy numbers of small subunit ribosomal DNA (SSU rDNA) (mean = red dot) of *T. suzukii* in single *D. suzukii* (SWD) larvae (orange), pupae (blue) and adults (green). The course of infection for second instar larvae (L2) inoculated with 4 × 10^4^ spores. Replicates/numbers (R/N): larvae 3–5: R/N = 2/4, pupae 5–8: R/N = 2/4, pupae 8–13: R/N = 3/5, adult 13–20: R/N = 3/6. GLM analysis for gamma distribution with log-link: *χ*^*2*^ = 14.014, *DF* = 3, *P* = 0.003, Tukey test: 3–5 and 13–20: *P* < 0.001, 5–8 and 13–20: *P* = 0.252, 8–13 and 13–20: *P* = 0.305. Different letters indicate statistical difference. Boxplot indicates the median logarithmic DNA quantity per individual (horizontal bar), mean (red dot), error bars (black hinge), asymptotic upper and lower confidence limits (red hinge) outliers (white dot). (**b**) Bright-field light microscopy of Giemsa-stained smears. Developmental stages of *T. suzukii* isolated from SWD larvae, pupae and adults at different time points post inoculation. SWD were inoculated in L2 larval stage with 4 × 10^4^ T*. suzukii* spores. (1) Larval stage (6 dpi). (2) Early pupal stage (8 dpi). (3) Pupal stages (10 dpi). (4) Late pupal stage (13 dpi). (5) Early adult stage (15 dpi). (6) Adult stage (17 dpi). Abbreviations: (M) meront containing two nuclei, (St) sporont, (Sp) spore, (dSt) dividing sporont, (Sb) sporoblast. Scale bar = 5 µm.

**Figure 3:**
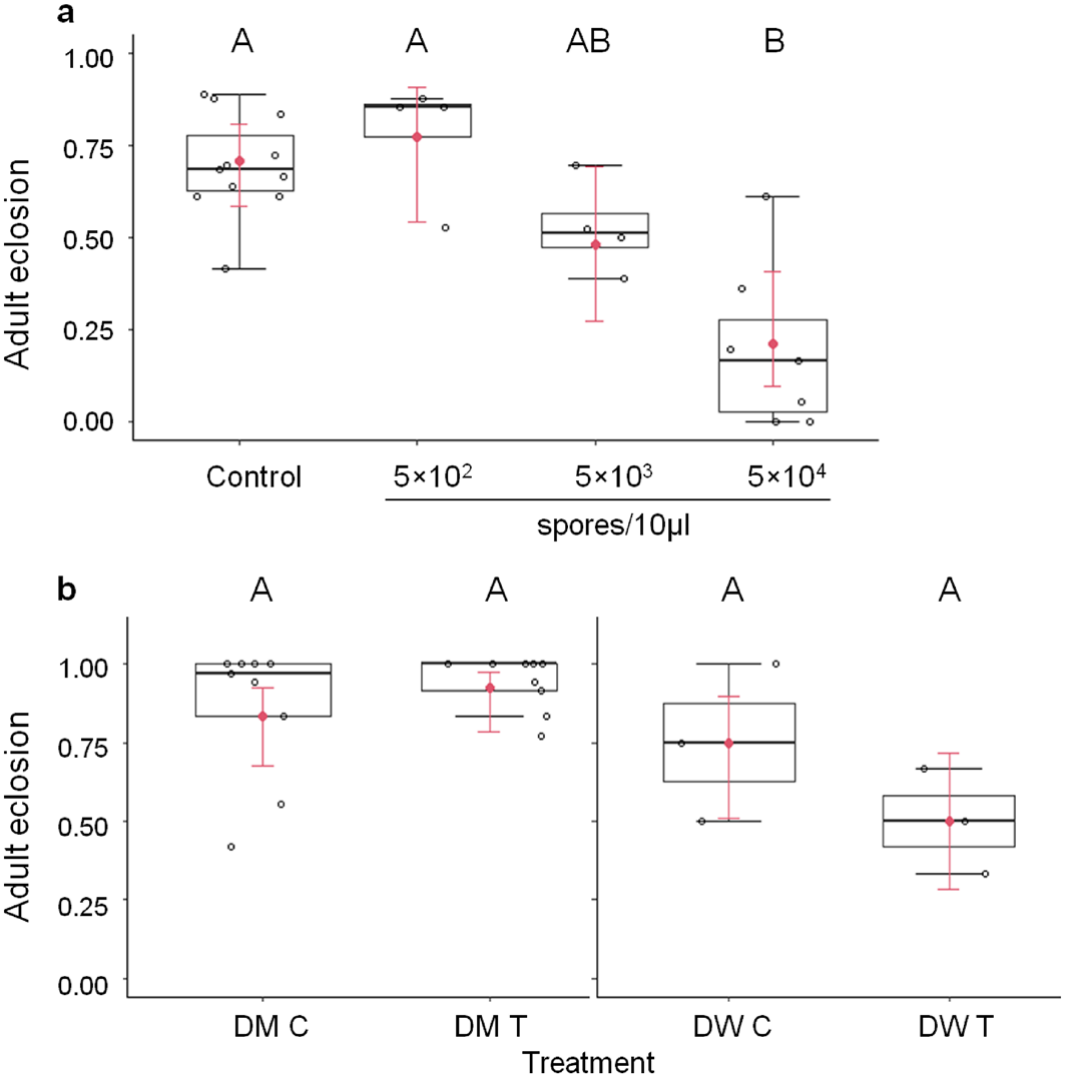
(**a**) *D. suzukii* adult eclosion (proportion, mean = red dot) at 19 dpi after exposure to different *T. suzukii* spore dosages (5 × 10^2^, 5 × 10^3^, 5 × 10^4^ spores/10 μl) in second instar (L2) larval stage and controls. Replicate/number (R/N): C = 11/408, 5 × 10^2^ = 4/180, 5 × 10^3^ = 4/204, 5 × 10^4^ = 7/264, *F* = 8.91, *DF* = 3, *P*(F) < 0.001. (**b**) Eclosion (proportion) of *D. melanogaster* (DM T) and *D.* *willistoni* (DW T) exposed to 5 × 10^4^ T*. suzukii* spores and corresponding controls (DM C, DW C) in L2 larval stage (replicates/total individuals (R/N), DM C: R/N = 9/287, DM T: R/N = 9/276, *F* = 1.361, *DF* = 1, *P*(F) = 0.261; DW control: R/N = 3/48, DW T: R/N = 3/48, *F* = 1.987, *DF* = 1, *P*(F) = 0.232). Different letters indicate statistical difference (see Fig. 2 for boxplot indication).

**Figure 4:**
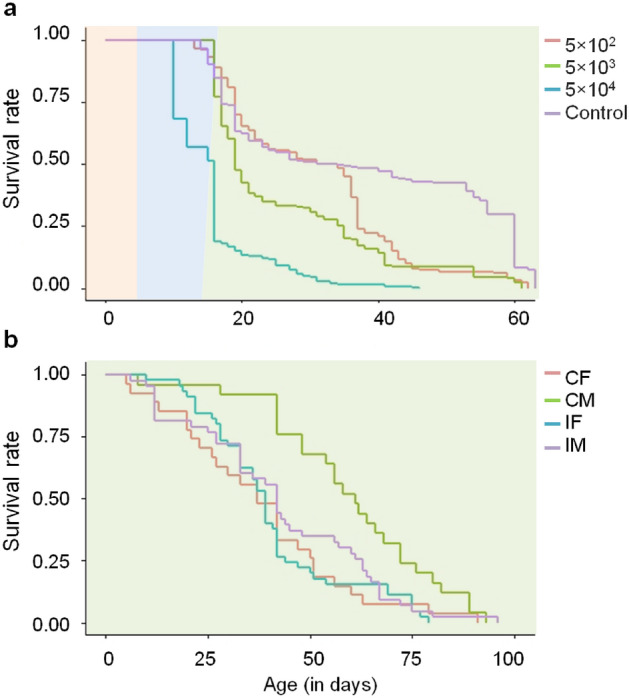
(**a**) Kaplan–Meier survival curves of *D. suzukii* (SWD), shown as complete lifespan from larval stage (day 0–6, orange field) to pupal stage (day 7–19, blue field) up to eclosion and death in adult stage (green field). Second instar larvae were either exposed to 5 × 10^2^ spores (red line, replicates/number (R/N) = 4/180), 5 × 10^3^ spores (green line, R/N = 4/204), 5 × 10^4^ spores (turquoise line, R/N = 7/264) or sterile water as control (purple line, R/N = 11/408). Log rank test: *χ*^2^ = 534, *DF* = 3, *P* < 0.001. (**b**) Kaplan–Meier survival curves of adult SWD inoculated with *T. suzukii* 3 days after emergence. Separated male and female SWD could either feed on sterile water as control (CF = control female, red, replicates/number (R/N) = 4/27; CM = control male, green, R/N = 4/25) or a spore suspension of 3 × 10^5^ T*. suzukii* spores (IF = inoculated female, turquoise, R/N = 4/45; IM = inoculated male, purple, R/N = 4/43). Log rank test: *χ*^2^ = 13.1 *DF* = 3, *P* = 0.004.

To study the infection process, SWD L2 larvae were inoculated with 4 × 10^4^ spores, followed by DNA extraction from larvae, pupae and adults for quantitative real-time PCR (qPCR) (Fig. 2a) and examination by microscopy (Fig. 2b). In third instars (L3) at 3–5 dpi, DNA copies equivalent to 1.33 ± 1.22 × 10^6^ (mean ± SE) extracted spores were recorded (Fig. 2a). In early pupal stage microsporidian DNA increased to 8.54 ± 7.12 × 10^6^ copies and was stable until late pupal stage (8–13 dpi) with 6.8 ± 2.97 × 10^6^ copies per SWD pupa. After the transition between late pupal stage to adulthood (13–20 dpi), the DNA amount increased by the factor of 20 in early adult stage with 13.3 ± 7.91 × 10^7^ copies per individual and was significantly increased compared to DNA copies at 3–5 dpi (GLM analysis for gamma distribution with log-link function: *χ*^*2*^ = 14.014, *DF* = 3, *P* = 0.003, Tukey test: 3–5 dpi–13–20 dpi: *P* < 0.001). Correspondingly, Giemsa-stained tissues of early larvae (6 dpi) revealed just a few free developmental stages consisting of meronts and sporonts (Fig. 2b-1). Early pupal stage (8 dpi) showed meronts containing two nuclei, diplokaryotic sporonts and sporonts close to division/ separation of nuclei (Fig. 2b-2). Later in pupal stage (10 dpi), few spores and predominantly (dividing) sporonts were observed (Fig. 2b-3. In late pupal stage (13 dpi), mainly spores could be found, but also sporoblasts (Fig. 2b-4). Adult stages (15–17 dpi) contained (Fig. 2b-5) mostly binucleate sporoblasts and single spores (Fig. 2b-6).

### Eclosion of inoculated SWD larvae

L2 larvae exposed to 5 × 10^4^ T*. suzukii* spores showed 71.2% reduced eclosion at 19 dpi (mean ± SE = 19.84 ± 8.41%) compared to the control (69.1 ± 4.14) (Fig. 3a). Eclosion at lower spore dosages did not statistically differ from the control group, though an intermediate mortality between those of the control and 5 × 10^3^ spores was observed for the treatment with 5 × 10^2^ spores GLM analysis for quasi-binomial model with over-dispersion: *F* = 8.91, *DF* = 3, *P*(F) < 0.001, Tukey test: Control—5 × 10^2^: *P* = 0.889, Control—5 × 10^3^: *P* = 0.226, Control—5 × 10^4^: *P* < 0.0001), (Fig. 3a).

For host range testing of cosmopolitan and exotic drosophilids, L2 larvae of *D*. *melanogaster* (DM, cosmopolitan) and *D*. *willistoni* (DW, exotic) were exposed to 5 × 10^4^ of *T. suzukii* spores/well. This spore concentration did not significantly reduce eclosion in DM and DW compared to their corresponding controls (GLM analysis for quasi-binomial model with over-dispersion: DM, *F* = 1.361, *DF* = 1, *P*(F) = 0.261; DW, *F* = 1.987, *DF* = 1, *P*(F) = 0.232), (Fig. 3b).

### Survival rates of inoculated SWD larvae and adults

Kaplan–Meier survival analyses were performed with individuals from the same experiment, which survived to the adult stage (Fig. 4a). Eclosion occurred from day 13 to day 19 as a result to some age differences among inoculated L2 larvae. Survival analysis was performed using log rank test with Bonferroni adjustment and revealed significant differences in the life span of SWD adults for every treatment compared to the control (Survival formula: *χ*^2^ = 534, *DF* = 3, *P* < 0.001) (Fig. 4a). It was striking that the survival curves of the control group and the group treated with 5 × 10^2^ spores were similar until day 35, then mortality increased in the treated group, indicating a delayed effect on the survival of adults when inoculated with very low spore concentration as larvae.

To study the effect of late infection initiation, newly eclosed SWD adults were treated with 3 × 10^5^ spores in 100 µl. In Kaplan–Meier analyses (Fig. 4b), survival rates of control males was significantly higher than infected males (*P* = 0.027), infected females (*P* = 0.001) and control females (*P* = 0.004), whereas control females had similar survival curves as infection treatments for both males and females (*χ*^2^ = 13.1, *DF* = 3, *P* = 0.004).

### Fecundity and fertility of SWD treated with *T. suzukii*

When SWD larvae were infected, the mean number (± SEM) of eggs oviposited per female was 135.97 ± 8.81 for the untreated control pairs (Table 1). Pairs with infected parental females deposited 51% less eggs compared to control. For pairs with infected parental males the reduction was 48% and approximately 71% reduction in oviposition when both parents were infected (Table 1). For pairs with at least one infected parent, oviposition differed significantly from the untreated control, indicating a strong effect of microsporidium infection on the fecundity of SWD (*χ*^2^ = 48.84, *DF* = 3, *P* < 0.05, Dunn’s test: F–C: *P* = 0.006, M-C: *P* = 0.019, MF-C: *P* < 0.001). The mean lifetime (Table 1) of pairs did not differ from each other (χ^2^ = 2.683, *DF* = 3, *P* = 0.44), indicating parental longevity was not a factor in the differences in fecundity and fertility among infected and control SWD.

**Table 1:** Lifetime, fecundity (oviposition) and fertility (offspring) of SWD pairs inoculated with *T. suzukii* spores at larval stage.

**ID**	Treatment	R/N	Life-time (days)	SEM	Eggs/pair/lifetime (§)	SEM	Eggs/pair/10 days (%) (§)	SEM	Offspring (§)	SEM	*P* (eggs/lifetime-offspring)	Sex ratio offspring ♀/♂	N (sex ratio)	P (sex ratio)
C	u♀ × u♂	3/40	35.9	2.1	135.9 (A)	8.8	61.3 (45.1) (A)	5.4	127.7 (A)	9.2	> 0.05, *T* = 1.09	0.49/0.51	3351	> 0.05, *χ*^2^ = 0.37
F	i♀ × u♂	3/8	32.8	3.5	65.4 (B)	22.8	30.4 (46.5) (B)	10.6	64.5 (B)	22.9	> 0.05, *T* = 0.03	0.47/0.53	179	> 0.05, *χ*^2^ = 0.45
M	u♀ × i♂	3/8	26.8	5.7	70.4 (B)	19.9	40.0 (56.8) (AB)	12.3	69.1 (AB)	19.9	> 0.05, *T* = 0.04	0.64/0.36	121	< 0.05, *χ*^2^ = 9.00
MF	i♀ × i♂	3/35	29.6	1.5	39.8 (B)	4.5	19.4 (48.7) (B)	2.8	32.2 (B)	4.8	> 0.05, *W* = 732.5	0.52/0.48	439	> 0.05, *χ*^2^ = 0.06
	*P*, test		> 0.05, *χ*^2^ = 2.68		< 0.001, *χ*^2^ = 48.84		< 0.001, *F* = 13.96		< 0.05, *χ*^2^ = 41.34					

Sex ratio of resulting offspring was determined after eclosion of progeny. ID = treatment (C = untreated control, F = female infected, M = male infected, MF = both infected), treatment: ♀ = female, ♂ = male, u = uninfected, i = infected, R/N = number of replicates and total pairs, (§) = significance letter (< 0.05), SEM = standard error of mean, N (sex ratio) = total number of offspring analysed for sex. Lifetime, eggs/pair/lifetime, eggs/pair/10 days, offspring are shown as means.

Comparing the number of eggs and resulting offspring within each treatment, no reduced hatching rates were observed (Table 1). Indeed, the number of viable offspring was very similar to number of eggs, thus the primary effect was on fecundity and not on fertility (*χ*^2^ = 41.336, *DF* = 3, *P* < 0.05, Dunn’s test (Bonferroni adjustment): F–C: *P* = 0.037, M-C: *P* = 0.073, MF-C: *P* < 0.001).

For control females, 45% of total eggs oviposited were laid in the first 10 days after eclosion (Table 1). Treatments of pairs including infected females differed significantly from the untreated control, but not pairs with only males infected (*F* = 13.96, *DF* = 3, *P*(F) < 0.001, Tukey HSD: F–C: *P* < 0.05, M-C: *P* > 0.05, MF-C: *P* < 0.001).

The sex ratio of the offspring was not affected in female treatments (*χ*^*2*^ = 0.45, *DF* = 1, *P* = 0.50) and trials with biparental (*χ*^*2*^ = 0.057, *DF* = 1, *P* = 0.81) infection compared to controls (*χ*^*2*^ = 0.37, *DF* = 1, *P* = 0.54); male to female ratio was close to 1:1 (Table 1). Male-only infection treatments revealed a sex-biased effect with significant more female offspring (*χ*^*2*^ = 9, *DF* = 1, *P* = 0.003) (Table 1). Microscopic inspection of 227 F1 individuals from transmission experiments with both infected parents (R/N = 2/18) revealed eight adult flies with a microsporidian infection (3.52%) 3 weeks after eggs laid.

When SWD adults were inoculated with *T. suzukii*, mean oviposition was approximately 30% less in microsporidian-treated groups (MF) compared to the control groups (C) (Table 2), but no significant difference between both treatments was noted. Also, the number of eclosed offspring recorded 18 days after oviposition did not differ among treatments (Table 2). In both treatments, fertility was about 15–20% lower than fecundity but this difference was not significant (Table 2).

**Table 2:** Lifetime, fecundity (oviposition) and fertility (offspring) of SWD pairs inoculated with *T. suzukii* spores at adult stage.

ID	Treatment	R/N	Eggs/pair/lifetime in days (§)	SEM	Offspring (§)	SEM	*P* (eggs-offspring)	Sex ratio offspring ♀/♂	N (sex ratio)	*P* (sex ratio)
C	u♀ × u♂	4/17	152.9 (A)	19.43	132.2 (A)	19.3	> 0.05, *T* = 0.75	0.51/0.49	1139	> 0.05, *χ*^2^ = 0.46
MF	i♀ × i♂	4/31	107.6 (A)	12.43	88.0 (A)	10.6	> 0.05, *W* = 573.5	0.54/0.46	1405	< 0.05, *χ*^2^ = 9.08
	*P*, test		0.059, *T* = − 1.96		0.055, *T* = − 2.01					

Sex ratio of resulting offspring was determined after eclosion of progeny. ID = treatment abbreviation (C = untreated control, MF = both infected), treatment: ♀ = female, ♂ = male, u = uninfected, i = infected, R/N = number of replicates and total pairs, (§) = significance letter (< 0.05), SEM = standard error of mean, N (sex ratio) = total number of offspring analysed for sex. Lifetime, eggs/pair/lifetime, eggs/pair/10 days, offspring are shown as mean.

The sex ratio of offspring from *T. suzukii* inoculated parents was shifted to female offspring (*χ*^*2*^ = 9.09, *DF* = 1, *P* = 0.003), control treatments showed a sex ratio close to 1:1 (*χ*^*2*^ = 0.46, *DF* = 1, *P* = 0.50) (Table 2). Inspection of 223 F1 adults derived from *T. suzukii* treatment did not show any infected offspring, indicating that the microsporidium is not vertically transmitted if infection occurs in adult stage. Microscopic inspection of 31 Microsporidia-treated pairs resulted in one male fly with an established *T. suzukii*-infection.

## Discussion

A novel microsporidium *T. suzukii* was discovered in 2015 in laboratory-reared SWD ^39^. In this study, the potential impact of *T. suzukii* infection on SWD larvae and adults has been investigated and significant concentration-dependent effects on the mortality as well as the eclosion, survival, lifetime fecundity and effects on offspring were analysed when L2 larvae were inoculated with the microsporidium.

We succeeded in measuring the infection progress in individual SWD inoculated in larval stage by qPCR, which allowed us following the replication cycle of *T. suzukii* upon emergence of flies. Within the first 13 days post inoculation, DNA copies of microsporidian SSU rDNA increased up to 1000-fold, indicating strong microsporidian proliferation and explaining low pupal survival and adult eclosion after exposure to a high number of spores. Although we counted a maximum of 10^6^ spores when dissecting flies, quantification of DNA copies suggested even up to 10^8^ spores per fly in late infection stages. We found high larval mortality, whereas eclosion declined dramatically (up to 70%) with increasing inoculated spore concentration. It has to be emphasized that the used amount of spores was experimentally motivated, but no data are available if such doses would be achieved in natural transmission scenarios when spores are released from dead individuals. SWD larvae surviving the *T. suzukii* infection would die during pupal or in early adult stages compared to controls. At high spore concentration, only 30% of flies survived the adult stage and these died within 40 days compared to 65 days in control. At low spore concentration, differences between the inoculated and control groups were not seen until day 35, when mortality increased. There was no evidence that *T. suzukii* has such strong effect on eclosion of other drosophilids when L2 larvae were inoculated. Compared to controls, eclosion of *D.* *willistoni* was reduced about 33% when larvae were inoculated with 5 × 10^4^ spores but *D. melanogaster* was not affected at all. Virulence of *T. suzukii* to these two drosophilids was low, but transmission efficiency as another important infection parameter^40^ was not determined for both in this study.

When adults of SWD were inoculated, male control flies had a significantly longer lifespan compared to other treatments. This effect could be due to the experimental design where one female and one male were kept together. As shown for other drosophilids, mating significantly reduces survival of females and the absence of rivals results in increased male lifespan^41–43^. The high larval and lower adult susceptibility of SWD are in line with findings from other microsporidia of drosophilids, such as *Tubulinosema ratisbonensis* and *Tubulinosema kingi*^23,44–49^.

Fecundity and fertility were significantly reduced after larval inoculation resulting in about 70% reduction of egg deposits in biparental infections, but not after adult treatment. Females appeared to be the mainly weakened, as SWD pairs with only females infected had the second highest reduction of egg deposits. One important fitness parameter is the oviposition rate within the first 10 days after adult emergence, when drosophilids are laying the majority of eggs compared to the rest of the lifetime^50^. SWD pairs inoculated with *T. suzukii* laid about 46–57% of the lifetime-eggs within the first 10 days, whereas untreated controls deposited 45%. Selection studies with continuous exposure of *D. melanogaster* to microsporidia showed increased longevity and higher early-life fecundity rates in selection lines when infected with the pathogen. In contrast, longevity of controls challenged with microsporidia was significantly shorter compared to the selection line, whereas the lifetime fecundity was higher in absence of the pathogen^51^. In our study, the sublethal concentrations for this experiment (single exposure of 10 μl with 1.5 × 10^4^ spores/μl) did not reduce adult fly survival. Hence, we observed no significantly higher oviposition than in controls within 10 dpi, comparable with results of earlier studies for *T. kingi*-infected *D. melanogaster*^44^. We found no change in fertility, all treatments showed only 10% reduced hatching of offspring assuming *T. suzukii* has no effect on fertility. No tissue-pathological evidence for *T. suzukii* infections in male gonads or sperms was found in a recent study, but sample size was low^39^. However, the combination of parental pairs with male-only infection (M: u♀ × i♂) resulted in reduced oviposition as well. We found a statistically significant shift to higher proportions of female offspring (0.64) compared to controls (0.49) in this experimental setup, suggesting a parthenogenetic effect due to microsporidian infection. Since this effect was found from only three infected pairs more replicates would be useful to further support this finding. To our knowledge, pathogen-induced parthenogenesis was never described for SWD, although *Wolbachia* bacteria are known to induce female-biased progeny for approximately 40 drosophilid species^52–56^. The number of progeny in male-only treatments was comparably low to female-only treatment. Previous studies reported unmated females producing unfertilized eggs have a reduced total number of progeny for some drosophilids species^57,58^.

The here discussed high larval and low adult infection effects were similar to previous results of Vijendravarma et al. on stage-specific susceptibility of *D. melanogaster* treated with *T. kingi*^47^. For invertebrate hosts, several resistance mechanisms after microsporidian exposure were reported, showing induced cellular immune response like encapsulation, melanisation, hemocyte production, and phagocytosis^65,66^. In Diptera (i.e. drosophilids and mosquitoes), microsporidia infection induced up-regulation of lysozym genes and AMP production^65^. In selection experiments with *D. melanogaster* challenged with the microsporidium *T. kingi* or the fungus *Beauveria bassiana*, similar effects were observed: Flies selected on the pathogens had increased fitness and higher intra-specific competitiveness under pathogen pressure compared to non-selected control lines in presence of pathogens^51,67^. Due to the relatedness of *T. kingi* and *T. suzukii*^39^, similar outcomes may hold for SWD*.* Hence, combined effects of potential resistance to *T. suzukii* as well as over-represented fitness costs with low adult susceptibility implicate that this microsporidium might be a difficult tool for SWD pest management. Above that, commercial field application of microsporidia for insect biocontrol is still a matter of debate. In the 1970s, *Antonospora* (*Paranosema*) *locustae* (formerly: *Nosema locustae*) was used for locust and grasshopper management^25^ but low and slow mortality, inefficiency for some target species, and insignificant efficacy resulted in an economic failure of this agent.

Furthermore, we conclude *T.* *suzukii* is not transmitted transovarially as offspring infection was rarely observed, but is horizontally transmitted via cadavers or frass. Transovum transmission is also possible. In former studies of the authors, *T. suzukii* spores were found inside maturing SWD ovaries of early adult stage females, possibly influencing egg development^39^. Spores were not found inside mature ovaries but infecting adipose tissue surrounding the ovaries, which can be transferred via egg deposition^39^. Other studies revealed transmission rates of 1–11% in *Drosophila* showing a similar transmission pattern for *Tubulinosema* microsporidia infecting closely related drosophilids^23,45^. In experiments where adults were inoculated, no oviposition reduction or vertical transmission was observed, suggesting a delayed infection process^59,60^.

In our experiments, *T. suzukii* had a very strong impact on longevity and fecundity of SWD in the lab when early larval stages were infected. Based on the negative effects, SWD infection could have a population reducing effect but adult flies are no suitable targets of *T. suzukii.* As this microsporidium was only found in a laboratory population so far, further field populations of SWD should be screened for natural occurrence of *T. suzukii* and other potential pathogens for biological control of this fruit damaging fly.

## Material and methods

### Insect host rearing

Microsporidia-free *D. suzukii* (SWD) flies were maintained in 30 × 30 × 30 cm cages (BUGDORM, MEGAVIEW SCIENCE CO., Taiwan) with water, a diet for adult flies (1 g brewer’s yeast and 1 g sucrose) and an artificial oviposition medium as described elsewhere (^39^, modified from Chabert, et al.^30^). The oviposition medium was replaced weekly. If larvae with synchronous development were needed, the medium was replaced every 4 h. Insect rearing and subsequent biotests were performed under the following conditions: 22 ± 1 °C, 50% relative humidity (r.H.), 16:8 h light:dark photoperiod. Microsporidia-free *D. melanogaster* (DM) and *D. willistoni* (DW) were maintained under same conditions as described for SWD.

### Preparation of *T. suzukii* spores for SWD inoculation

*T. suzukii* was first isolated from a *D. suzukii* rearing originating from flies collected in Oregon, USA^38^. Adult *D. suzukii* were homogenized with a micro pestle, dissolved in distilled sterile water and filtered through four layers of gauze, then additionally filtered through a cotton filter disc with 12–15 μm particle retention (Grade 1288, Ø 90 mm, SARTORIUS AG, Göttingen, Germany). Spores were precipitated by centrifugation at 10,000×*g* (Centrifuge 5424 R, EPPENDORF, Hamburg, Germany) and resuspended in 500 μl sterile tap water. Spore concentration and purity was determined with a Thoma hemocytometer under phase contrast microscopy (DMRB, LEICA, Wetzlar, Germany), followed by dilution in sterile tap water to final concentrations as required for subsequent biotests.

### Preparation of standards in real-time quantitative PCR (qPCR)

*T. suzukii* spores were extracted from adult SWD carrying an infection with spores (about 3 weeks after initial inoculation of L2 larvae with 10 μl spore suspension containing 1.5 × 10^4^ T*. suzukii* spores). To produce standard curves, ten flies were homogenized with a micro pestle. About 1 × 10^7^ spores were purified with one filtration step through four layers of gauze mesh and a final purification with Percoll. For this purpose, 400 μl spore suspension was overlaid on 1.6 ml 75% Percoll (MERCK, Darmstadt, Germany) dissolved in 1 × PBS in a 2-ml reaction tube and spun down for 20 min at 12,900×*g* and 15 °C in an EPPENDORF centrifuge (5424R, EPPENDORF, Hamburg, Germany). The spores formed a band close to the bottom of the reaction tube. The spore band was washed twice in 1 × PBS at 15,000×*g* for 5 min, and the resulting pellet was resuspended in distilled water. Afterwards, spores were inspected for purity under phase contrast microscopy (DMRB, LEICA, Wetzlar, Germany). For preparation of a qPCR standard, serial dilutions of purified spores were prepared with 10^1^–10^6^ spores in 100 μl distilled water.

### SWD inoculation and DNA extraction for qPCR

L2 larvae of SWD were exposed to 10 μl spore suspension with 4 × 10^4^ spores in a microtiter plate overlaid on 440 μl pureed apple. Every 2–3 days three larvae and later pupae or adults were removed and euthanized with ethyl-acetate and surface sterilized with 0.05% sodium hypochlorite. One individual was examined visually for infection by light microscopy with 400 × magnification (DMRB, LEICA, Wetzlar, Germany) and a smear was stained with modified Giemsa-stain according to Eberle et al.^61^. Two larvae per replicate were used for genomic DNA extraction as described above and then qPCR. This was repeated for adult SWD which were starved for 3 h followed by bulk feeding for 18 h with a spore suspension containing in total 5 × 10^5^ T*. suzukii* spores mixed with blue food colour (modified droplet feeding method from Hughes and Wood^62^). To each group of ten flies, 10 μl spore suspension were provided. The time frame for euthanizing four flies per replicate (day 3, 5, 10, 18, 28, 38) was longer than for larval inoculation, as infection was sometimes delayed in adults. Two flies were prepared for microscopy and Giemsa-staining and two other were used for genomic DNA extraction after surface sterilization with sodium hypochlorite.

Sample and standard spore preparations (see “Preparation of standards in real-time quantitative PCR (qPCR)”) were centrifuged at 15,000×*g* for 10 min. The spore pellet was dissolved in 200 μl CTAB lysis buffer (APPLICHEM, Darmstadt, Germany). Following addition of 200 mg of glass beads (0.25–0.5 mm diameter, ROTH, Karlsruhe, Germany) spores were broken by bead beating in a tissue disrupter at 24 MHz for 1 min (MP FASTPREP-24 Tissue and Cell Homogenizer, MP BIOMEDICALS, Eschwege, Germany). Lysis was performed by adding 2 μl proteinase K (200 ng/μl, BIORON GMBH, Römerberg, Germany) and incubating samples at 56 °C on a thermo shaker at 250 rpm for 18 h. DNA was extracted with a two-step phenol–chloroform extraction with 25:24:1 phenol:chloroform:isoamyl alcohol. DNA preparations were washed twice in chloroform and finally subjected to ethanol precipitation (96% ethanol). DNA pellets were dissolved in 30 μl distilled water. Quantitative PCR reactions to amplify small ribosomal subunit DNA (SSU rDNA) were carried out with 2 μl of DNA dissolved in distilled water and Maxima SYBR Green qPCR Master Mix (THERMO FISHER SCIENTIFIC GMBH, Darmstadt, Germany) using 12.5 μl Master Mix, 1 μl 10 mM forward primer Tn37F (5′-CGAAGATTTAGCCATGCATGCT-3′) and 1 μl 10 mM reverse primer Tn562R (5′´-CCGCTTCGAATATAAGCATTGA-3′) ^39^ and 8.5 μl Nuclease-free water per reaction with following reaction conditions: 94 °C for 3 min initial denaturation, followed by 35 cycles of 94 °C for 45 s, 50 °C for 30 s, 72 °C for 90 s and stepwise temperature increase from 50 °C to 94 °C in 0.5 °C every 5 s (CFX96 TOUCH Real Time PCR Systems, BIO-RAD LABORATORIES GMBH, Feldkirchen, Germany).

### Larval inoculation for lethal concentration (LC_50_), survival and fecundity tests

The cavities of a 96-well microtiter plate (GREINER BIO-ONE GMBH, Frickenhausen, Germany) were filled with 440 μl pureed apple overlaid with 10 μl spore suspension (concentrations for each experiment are listed below). A single L2 larva (3 days-old) was placed in each well the microtiter plate. Four experimental set ups were conducted:

- LC_50_ determination: application of five different spore concentrations in logarithmic scale (10^1^ to 10^5^ spores per μl) to L2 larvae in a single microtiter plate. For each concentration one microtiter plate was prepared to avoid contamination and spill-over of larvae to another treatment. Two to nine independent replicates were performed.
- Eclosion and survival experiments: application of either 5 × 10^2^, 5 × 10^3^ or 5 × 10^4^/10 μl T*.* *suzukii* spores to each larva in one single microtiter plate. For each concentration one microtiter plate was prepared to avoid contamination. Four to eleven replicates were performed. For each independent replicate, a new spore suspension was prepared.
- Fecundity tests: application of 1.5 × 10^4^/10 μl T*.* *suzukii* spores to each larva in a single microtiter plate.
- Host range testing with *D. melanogaster* and *D.* *willistoni*: application of 5 × 10^4^/10 μl T*. suzukii* spores to each one L2 larva of one species in a single microtiter plate. Three to nine replicates were prepared.

Untreated controls were prepared in separate microtiter plates and contained the equivalent amount (10 μl) of sterile tap water overlaid on 440 μl pureed apple. All implemented microtiter plates of one replicate for infection treatment and controls were prepared identically using the same batch of L2 larvae and kept under same rearing conditions.

### Experimental design for LC_50_, eclosion and survival tests

Microtiter plates containing inoculated larvae were transferred into cylindrical cages (30 cm height, 25 cm diameter, closed with a nylon membrane) containing a water source, adult diet and oviposition medium (see 2.1) were changed twice a week. Mortality in LC_50_ tests until 18 days post inoculation (dpi), eclosion until 19 dpi and survival of adults until 63 dpi were recorded daily until all SWD died. The LC_50_ at 18 dpi was calculated using probit analysis. Mortality data were corrected for control mortality by Abbott formula^63^.

### Experimental design for fecundity tests

For fecundity experiments, inoculated larvae pupated and eclosed and the adults were separated into male and female groups directly after eclosion for 3 days to avoid premature mating. One 3-day-old naive male and female adult were then placed together for mating and oviposition They were held in boxes (6 cm height, 10 cm diameter) containing a Petri dish (3 cm diameter) with oviposition medium prepared as described in 2.1. After 48 h, the Petri dish with oviposition medium was replaced and eggs were counted from the oviposition medium. The oviposition medium was then placed in a separate box and eggs were maintained until hatch. This procedure was repeated every 2 days until the last SWD pair inoculated with *T. suzukii* died. Number of hatched offspring was determined 18 days after oviposition and sex ratios were recorded. Treated flies were post-hoc inspected for established infections and assigned to different groups in the analyses: MF: i♀ × i♂ = female and male infected, F: i♀ × u♂ = female infected/male uninfected, M: u♀ × i♂ = female uninfected/male infected. Transmission was examined in two separate trials (18 pairs) with both infected parents. Oviposition medium was changed every 2 days as described above and the oviposition rate was recorded. Eggs were transferred to fresh medium for development. Eggs were not surface sterilized to avoid manipulation of the respiratory filaments resulting in higher mortality. Experiments were carried out in an incubator (RUMED 3501, RUBARTH, Laatzen, Germany) under rearing conditions: 22 ± 1 °C, 60% rH, 16:8 h light–dark photoperiod.

### Inoculation of adults and design of survival and fecundity experiments

SWD adults at 3 days post-eclosion (survival experiment) or one day post-eclosion (fecundity experiment) were placed together in groups of four (survival experiment) or separated into groups of male-only and female-only groups (fecundity experiment) into a plastic box where they were starved for 3 h followed by bulk feeding with 100 μl tap water droplets containing in total 3 × 10^5^ T*. suzukii* spores mixed with blue food colour over night (about 18 h) (modified droplet feeding method from Hughes and Wood^62^). Flies with a blue abdomen were selected for the experiments, whereby one male and one female were placed together in a cage (containing diet and water). For each independent replicate, a new spore suspension was prepared. Four replicates were prepared in total. In survival experiments, daily survival was recorded until all adults died. For fecundity experiments, egg deposition was recorded every 2 days and number of hatched offspring was determined 18 days post oviposition. Treated flies were post-hoc microscopically inspected to determine infection status.

### Statistical analyses

Estimation of the median lethal concentration (LC_50_) and slope of the concentration–mortality curve were calculated by probit analysis using TOXRAT software^64^. All other statistical analyses were conducted with R version 3.5.1 (2018–07–02). For test of normal distribution, Shapiro Wilk test was chosen with α = 0.05 level of significance. For qPCR DNA expression data and adult eclosion assays general linear models (GLM) were for gamma and quasi-binomial distributed data were used respectively. Numbers of independent replicates (R) and sample size (N) are given in the Results part. For all statistical tests a level of significance of α = 0.05 was applied if not indicated otherwise.

### Author statements

The authors confirm that all methods described in the submitted manuscript "Infection effects of the new microsporidian species *Tubulinosema suzukii* on its host *Drosophila suzukii"* were carried out in accordance with relevant guidelines and regulations in the manuscript.

The authors confirm that all methods and experimental protocols used in the submitted manuscript "Infection effects of the new microsporidian species *Tubulinosema suzukii* on its host *Drosophila suzukii"* were approved by the Julius Kühn-Institut, Federal Research Centre for Cultivated Plants.

## References

[1] Capella-Gutiérrez S, Marcet-Houben M, Gabaldon T (2012). Phylogenomics supports microsporidia as the earliest diverging clade of sequenced fungi. BMC Biol..

[2] Corsaro D (2019). Filling gaps in the microsporidian tree: rDNA phylogeny of *Chytridiopsis typographi* (Microsporidia: Chytridiopsida). Parasitol. Res..

[3] Corsaro D (2016). Molecular identification of *Nucleophaga terricolae* sp. nov. (Rozellomycota), and new insights on the origin of the Microsporidia. Parasitol. Res..

[4] James TY (2006). Reconstructing the early evolution of Fungi using a six-gene phylogeny. Nature.

[5] Sprague, V. & Becnel, J. J. in *The Microsporidia and Microsporidiosis* (eds M. Wittner & L. M. Weiss) 517–530 (ASM Press, 1999).

[6] Dunn AM, Terry RS, Smith JE (2001). Transovarial transmission in the microsporidia. Adv. Parasitol..

[7] Goertz D, Hoch G (2008). Vertical transmission and overwintering of Microsporidia in the gypsy moth, *Lymantria dispar*. J. Invertebr. Pathol..

[8] Becnel, J. J. & Andreadis, T. G. in *Microsporidia: Pathogens of Opportunity* (eds L. M. Weiss & J. J. Becnel) 521–570 (Wiley, 2014).

[9] Kellen WR, Lindegren JE (1971). Modes of transmission of *Nosema plodiae* Kellen and Lindegren, a pathogen of *Plodia interpunctella* (Hübner). J. Stored Prod. Res..

[10] Vávra, J. & Larsson, R. J. in *Microsporidia: Pathogens of Opportunity* (eds L. M. Weiss & J. J. Becnel) 1–70 (Wiley, 2014).

[11] Mudasar M, Mathivanan V, Shah GN, Mir GM, Selvisabhanayakam M (2013). Nosemosis and its effect on performance of honey bees: A review. Int. J. Pharm. Bio. Sci..

[12] Wolf S, McMahon DP, Lim KS, Pull CD, Clark SJ, Paxton RJ, Osborne JL (2014). So near and yet so far: Harmonic radar reveals reduced homing ability of *Nosema* infected honeybees. PLoS ONE.

[13] Naug D, Gibbs A (2009). Behavioral changes mediated by hunger in honeybees infected with *Nosema ceranae*. Apidologie.

[14] Dussaubat C (2013). Flight behavior and pheromone changes associated to *Nosema ceranae* infection of honey bee workers (*Apis mellifera*) in field conditions. J. Invertebr. Pathol..

[15] Goblirsch M, Huang ZY, Spivak M (2013). Physiological and behavioral changes in honey bees (*Apis mellifera*) induced by *Nosema ceranae* infection. PLoS ONE.

[16] Lipsitch M, Nowak MA, Ebert D, May RM (1995). The population dynamics of vertically and horizontally transmitted parasites. Proc. R. Soc. Lond. B.

[17] Goertz D, Solter LF, Linde A (2007). Horizontal and vertical transmission of a *Nosema* sp. (Microsporidia) from *Lymantria dispar* (L.) (Lepidoptera: Lymantriidae). J. Invertebr. Pathol..

[18] Kellen WR, Chapman HC, Clark TB, Lindegren JE (1965). Host-parasite relationships of some *Thelohania* from mosquitoes (Nosematidae: Microsporidia). J. Invertebr. Pathol..

[19] Dunn AM, Smith JE (2001). Microsporidian life cycles and diversity: the relationship between virulence and transmission. Microbes Infect..

[20] Terry RS (2004). Widespread vertical transmission and associated host sex–ratio distortion within the eukaryotic phylum Microspora. Proc. R. Soc. Lond. B.

[21] Mercer C, Wigley P (1987). A microsporidian pathogen of the poroporo stem borer, *Sceliodes cordalis* (Dbld)(Lepidoptera: Pyralidae): Effects on adult reproductive success. J. Invertebr. Pathol..

[22] Bauer LS, Nordin GL (1989). Effect of *Nosema fumiferanae* (Microsporida) on fecundity, fertility, and progeny performance of *Choristoneura fumiferana* (Lepidoptera: Tortricidae). Environ. Entomol..

[23] Futerman P (2006). Fitness effects and transmission routes of a microsporidian parasite infecting *Drosophila* and its parasitoids. Parasitology.

[24] Goertz D, Golldack J, Linde A (2008). Two different and sublethal isolates of *Nosema lymantriae* (Microsporidia) reduce the reproductive success of their host, *Lymantria dispar*. Biocontrol Sci. Technol..

[25] Lockwood JA, Bomar CR, Ewen AB (1999). The history of biological control with *Nosema locustae*: Lessons for locust management. Int. J. Trop. Insect Sci..

[26] Kiritani, K. & Yamamura, K. in *Invasive Species: Vectors and Management Strategies.* (ed J. Carlton) 44–67 (Island Press, 2003).

[27] Walsh DB (2011). *Drosophila suzukii* (Diptera: Drosophilidae): invasive pest of ripening soft fruit expanding its geographic range and damage potential. J. Integr. Pest Manage..

[28] Cini A, Ioriatti C, Anfora G (2012). A review of the invasion of *Drosophila suzukii* in Europe and a draft research agenda for integrated pest management. Bull. Insectol..

[29] Tochen S (2014). Temperature-related development and population parameters for *Drosophila suzukii* (Diptera: Drosophilidae) on cherry and blueberry. Environ. Entomol..

[30] Chabert S, Allemand R, Poyet M, Eslin P, Gibert P (2012). Ability of European parasitoids (Hymenoptera) to control a new invasive Asiatic pest, *Drosophila suzukii*. Biol. Control.

[31] Gabarra R, Riudavets J, Rodríguez G, Pujade-Villar J, Arnó J (2015). Prospects for the biological control of *Drosophila suzukii*. Biocontrol.

[32] Cuthbertson AGS, Audsley N (2016). Further screening of entomopathogenic fungi and nematodes as control agents for *Drosophila suzukii*. Insects.

[33] Woltz JM, Donahue KM, Bruck DJ, Lee JC (2015). Efficacy of commercially available predators, nematodes and fungal entomopathogens for augmentative control of *Drosophila suzukii*. J. Appl. Entomol..

[34] Haye T (2016). Current SWD IPM tactics and their practical implementation in fruit crops across different regions around the world. J. Pest. Sci..

[35] Biganski S, Jehle JA, Kleespies RG (2018). Bacillus thuringiensis serovar israelensis has no effect on *Drosophila suzukii* Matsumura. J. Appl. Entomol..

[36] Carrau T, Hiebert N, Vilcinskas A, Lee K-Z (2018). Identification and characterization of natural viruses associated with the invasive insect pest *Drosophila suzukii*. J. Invertebr. Pathol..

[37] Medd NC (2017). The virome of *Drosophila suzukii*, an invasive pest of soft fruit. BioRxiv.

[38] Kaur R, Siozios S, Miller WJ, Rota-Stabelli O (2017). Insertion sequence polymorphism and genomic rearrangements uncover hidden Wolbachia diversity in *Drosophila suzukii* and *D. subpulchrella*. Sci. Rep..

[39] Biganski, S. *et al.* Molecular and morphological characterisation of a novel microsporidian species, *Tubulinosema suzukii*, infecting *Drosophila suzukii* (Diptera: Drosophilidae). *J. Invertebr. Pathol.* 107440 (2020). 10.1016/j.jip.2020.107440 32663547

[40] Anderson RM, May RM (1982). Coevolution of hosts and parasites. Parasitology.

[41] Aigaki T, Ohba S (1984). Effect of mating status on *Drosophila virilis* lifespan. Exp. Gerontol..

[42] Partridge L, Green A, Fowler K (1987). Effects of egg-production and of exposure to males on female survival in *Drosophila melanogaster*. J. Insect Physiol..

[43] Bretman A, Westmancoat JD, Gage MJ, Chapman T (2013). Costs and benefits of lifetime exposure to mating rivals in male *Drosophila melanogaster*. Evolution.

[44] Armstrong E, Bass LK (1989). *Nosema kingi*: Effects on fecundity, fertility, and longevity of *Drosophila melanogaster*. J. Exp. Zool..

[45] Armstrong E (1976). Transmission and infectivity studies on *Nosema kingi* in *Drosophila willistoni* and other Drosophilids. Z. Parasitenkd..

[46] Armstrong E, Bass L, Staker K, Harrell L (1986). A comparison of the biology of a *Nosema* in *Drosophila melanogaster* to *Nosema kingi* in *Drosophila willistoni*. J. Invertebr. Pathol..

[47] Vijendravarma RK, Godfray HCJ, Kraaijeveld AR (2008). Infection of *Drosophila melanogaster* by *Tubulinosema kingi*: Stage-specific susceptibility and within-host proliferation. J. Invertebr. Pathol..

[48] Niehus S, Giammarinaro P, Liégeois S, Quintin J, Ferrandon D (2012). Fly culture collapse disorder: Detection, prophylaxis and eradication of the microsporidian parasite *Tubulinosema ratisbonensis* infecting *Drosophila melanogaster*. Fly.

[49] Franchet A, Niehus S, Caravello G, Ferrandon D (2019). Phosphatidic acid as a limiting host metabolite for the proliferation of the microsporidium *Tubulinosema ratisbonensis* in *Drosophila* flies. Nat Microbiol.

[50] Robertson FW, Sang JH (1944). The ecological determinants of population growth in a *Drosophila* culture. I. Fecundity of adult flies. Proc. R. Soc. Lond. B.

[51] Vijendravarma RK, Kraaijeveld AR, Godfray HCJ (2009). Experimental evolution shows *Drosophila melanogaster* resistance to a microsporidian pathogen has fitness costs. Evolution.

[52] Rousset F, Bouchon D, Pintureau B, Juchault P, Solignac M (1992). *Wolbachia* endosymbionts responsible for various alterations of sexuality in arthropods. Proc. R. Soc. Lond. B.

[53] Saeed N, Battisti A, Martinez-Sañudo I, Mori N (2018). Combined effect of temperature and *Wolbachia* infection on the fitness of *Drosophila suzukii*. Bull. Insectol..

[54] Hamm CA (2014). Wolbachia do not live by reproductive manipulation alone: infection polymorphism in *Drosophila suzukii* and *D. subpulchrella*. Mol. Ecol..

[55] Mazzetto F, Gonella E, Alma A (2015). *Wolbachia* infection affects female fecundity in *Drosophila suzukii*. Bull. Insectol..

[56] Hurst, G. D., Johnson, A. P., vd Schulenburg, J. H. G. & Fuyama, Y. Male-killing *Wolbachia* in *Drosophila*: a temperature-sensitive trait with a threshold bacterial density. *Genetics* **156**, 699–709 (2000). 10.1093/genetics/156.2.699 PMC1461301 11014817

[57] Markow TA (2013). Parents without partners: *Drosophila* as a model for understanding the mechanisms and evolution of parthenogenesis. G3.

[58] Wolfner MF (2002). The gifts that keep on giving: physiological functions and evolutionary dynamics of male seminal proteins in *Drosophila*. Heredity.

[59] Blaser M, Schmid-Hempel P (2005). Determinants of virulence for the parasite *Nosema whitei* in its host *Tribolium castaneum*. J. Invertebr. Pathol..

[60] Solter, L. F. in *Microsporidia: Pathogens of Opportunity* (eds L. M. Weiss & J. J. Becnel) 165–194 (Wiley, 2014).

[61] Eberle, K. E., Wennmann, J. T., Kleespies, R. G. & Jehle, J. A. in *Manual of Techniques in Invertebrate Pathology* (ed L. A. Lacey) 15–74 (Academic Press, 2012).

[62] Hughes P, Wood H (1981). A synchronous peroral technique for the bioassay of insect viruses. J. Invertebr. Pathol..

[63] Abbott W (1925). A method of computing the effectiveness of an insecticide. J. Econ. Entomol..

[64] Software for the statistical analysis of biotests (ToxRat GmbH, Alsdorf, Germany, 2003).

[65] Pan G, Bao J, Ma Z, Song Y, Han B, Ran M, Li C, Zhou Z (2018). Invertebrate host responses to microsporidia infections. Dev. Comp. Immunol..

[66] Roxström-Lindquist K, Terenius O, Faye I (2004). Parasite-specific immune response in adult *Drosophila melanogaster*: A genomic study. EMBO Rep..

[67] Kraaijeveld AR, Godfray HCJ (2008). Selection for resistance to a fungal pathogen in *Drosophila melanogaster*. Heredity.

